# Gamma-glutamyl-leucine levels are causally associated with elevated cardio-metabolic risks

**DOI:** 10.3389/fnut.2022.936220

**Published:** 2022-11-24

**Authors:** Qiong Wu, Jiankang Li, Jinghan Zhu, Xiaohui Sun, Di He, Jun Li, Zongxue Cheng, Xuhui Zhang, Yuying Xu, Qing Chen, Yimin Zhu, Maode Lai

**Affiliations:** ^1^Department of Epidemiology and Biostatistics, Zhejiang University School of Medicine, Hangzhou, Zhejiang, China; ^2^Department of Respiratory Diseases, Sir Run Run Shaw Hospital, Zhejiang University School of Medicine, Hangzhou, Zhejiang, China; ^3^Department of Epidemiology and Biostatistics, School of Public Health, Hangzhou Normal University, Hangzhou, China; ^4^Institute of Medical Research, Northwestern Polytechnical University, Xi’an, China; ^5^The Second School of Clinical Medicine, Southern Medical University, Guangzhou, China; ^6^Department of Epidemiology and Biostatistics, School of Public Health, Zhejiang Chinese Medical University, Hangzhou, China; ^7^Hangzhou Center for Disease Control and Prevention, Hangzhou, China; ^8^Affiliated Hangzhou Center of Disease Control and Prevention, School of Public Health, Zhejiang University, Hangzhou, China; ^9^Zhejiang Provincial Centers for Disease Control and Prevention, Hangzhou, China; ^10^Cancer Center, Zhejiang University, Hangzhou, China; ^11^Key Laboratory of Disease Proteomics of Zhejiang Province, Department of Pathology, School of Medicine, Zhejiang University, Hangzhou, China; ^12^State Key Laboratory of Natural Medicines, School of Basic Medical Sciences and Clinical Pharmacy, China Pharmaceutical University, Nanjing, China

**Keywords:** Gamma-glutamyl-leucine, metabolic risk factors, GWAS, metabolic syndrome, Mendelian randomization

## Abstract

**Objective:**

Gamma-glutamyl dipeptides are bioactive peptides involved in inflammation, oxidative stress, and glucose regulation. Gamma-glutamyl-leucine (Gamma-Glu-Leu) has been extensively reported to be associated with the risk of cardio-metabolic diseases, such as obesity, metabolic syndrome, and type 2 diabetes. However, the causality remains to be uncovered. The aim of this study was to explore the causal-effect relationships between Gamma-Glu-Leu and metabolic risk.

**Materials and methods:**

In this study, 1,289 subjects were included from a cross-sectional survey on metabolic syndrome (MetS) in eastern China. Serum Gamma-Glu-Leu levels were measured by untargeted metabolomics. Using linear regressions, a two-stage genome-wide association study (GWAS) for Gamma-Glu-Leu was conducted to seek its instrumental single nucleotide polymorphisms (SNPs). One-sample Mendelian randomization (MR) analyses were performed to evaluate the causality between Gamma-Glu-Leu and the metabolic risk.

**Results:**

Four SNPs are associated with serum Gamma-Glu-Leu levels, including rs12476238, rs56146133, rs2479714, and rs12229654. Out of them, rs12476238 exhibits the strongest association (Beta = −0.38, S.E. = 0.07 in discovery stage, Beta = −0.29, S.E. = 0.14 in validation stage, combined *P*-value = 1.04 × 10^–8^). Each of the four SNPs has a nominal association with at least one metabolic risk factor. Both rs12229654 and rs56146133 are associated with body mass index, waist circumference (WC), the ratio of WC to hip circumference, blood pressure, and triglyceride (5 × 10^–5^ < *P* < 0.05). rs56146133 also has nominal associations with fasting insulin, glucose, and insulin resistance index (5 × 10^–5^ < *P* < 0.05). Using the four SNPs serving as the instrumental SNPs of Gamma-Glu-Leu, the MR analyses revealed that higher Gamma-Glu-Leu levels are causally associated with elevated risks of multiple cardio-metabolic factors except for high-density lipoprotein cholesterol and low-density lipoprotein cholesterol (*P* > 0.05).

**Conclusion:**

Four SNPs (rs12476238, rs56146133, rs2479714, and rs12229654) may regulate the levels of serum Gamma-Glu-Leu. Higher Gamma-Glu-Leu levels are causally linked to cardio-metabolic risks. Future prospective studies on Gamma-Glu-Leu are required to explain its role in metabolic disorders.

## Introduction

Cardiovascular disease (CVD) is the leading cause of mortality worldwide, responsible for 32% of global deaths (1). The increasing burden of cardio-metabolic risk is closely associated with CVD (2). Metabolic syndrome (MetS) is a composite of a series of cardio-metabolic risk factors, including central obesity, hypertension, dyslipidemia, and hyperglycemia (3). Exploring the pathophysiology of MetS and its related metabolic disorders could provide novel insights to prevent the progression of CVD.

Gamma-glutamyl dipeptides are a family of bioactive peptides containing gamma-glutamyl residues and amino acids that can be derived from dietary consumption (including cheese, soy sauce, edible beans, and so on) or produced from Gamma-glutamyl-cysteine synthetase (γ-GCS) and Gamma-glutamyl transferase (γ-GGT) metabolism in humans and microorganisms (4–6). Emerging evidence has demonstrated that Gamma-glutamyl dipeptides are involved in diverse bioactivities, including inflammatory activities, oxidative stress, and glucose metabolism *via* activating calcium-sensing receptors (CasR) in different organs (7–10). As a G-protein-coupled receptor, CaSR can modulate a large variety of cellular processes that are associated with cardiovascular health, such as modulation of insulin secretion, release of nitric oxide, upregulation of apoptosis and cell proliferation, and activation of the NLRP3 inflammasome (11). In epidemiological studies, disturbed Gamma-glutamyl dipeptide levels have also been implicated in multiple diseases, including obesity, MetS, type 2 diabetes, non-alcoholic fatty liver diseases, and CVDs (12–16). Our previous targeted metabolomics study also revealed that one of the Gamma-glutamyl dipeptides, Gamma-glutamyl-leucine (Gamma-Glu-Leu) levels, were significantly higher in patients with MetS (17). Inconsistently, Zheng et al. reported multiple Gamma-glutamyl dipeptides including Gamma-Glu-Leu to be associated with lower alcohol consumption (18). By using different Gamma-glutamyl dipeptides to establish a metabolite score, they found this score negatively associated with inflammation biomarkers and the incidence of CVDs (18). Given that most prior studies were based on cross-sectional designs, it still could not be decided whether the associations between Gamma-glutamyl dipeptides and the CVD-related metabolic factors were contributed by confounding factors, reverse causality, or real causal effectors. Much effort is required to determine the causal relationships between Gamma-glutamyl dipeptides and the metabolic risk.

Using genetic variants as instrumental variables (IVs), the Mendelian randomization (MR) approach has been widely applied to infer the causal-effect relationships (19, 20). Since genotype is assorted randomly at conception and the randomization process is emulated within observational studies, it is less likely to be affected by potential confounding factors or reverse causations (21, 22). As an intermediate phenotype between genetics and clinical disease endpoints, the metabolite phenotype has been found to have a high heritability (23). Genome-wide association studies for metabolites (mGWAS) have identified a hundred gene loci regulating metabolite levels (met-QTL) (24–26). On the one hand, these identified gene loci improved our understanding of the role of metabolites in disease etiologies; on the other hand, by using these identified genetic variants to proxy levels of metabolites, several previous studies have found that plasma metabolite levels, such as branched-chain amino acids and 2-hydroxybutyric acid, were causally associated with obesity and type 2 diabetes (21, 27).

Therefore, we hypothesize that the levels of Gamma-Glu-Leu are associated with single-nucleotide polymorphisms (SNPs) across the whole genome and that genetically determined Gamma-Glu-Leu has a causal effect on the cardio-metabolic risks. In this study, we aimed to identify the genetic variants associated with circulating levels of Gamma-Glu-Leu and to determine the causal relationships between the genetically proxied levels of Gamma-Glu-Leu and the cardio-metabolic factors using a MR approach.

## Materials and methods

### Study population

**FIGURE 1:**
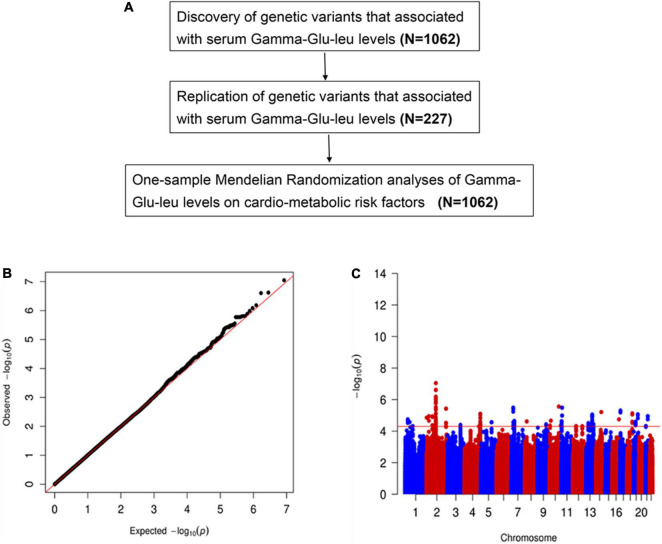
**(A)** The study overview. The cardio-metabolic risk factors include body mass index, waist circumference, waist circumference to hip circumference ratio, total triglyceride, total cholesterol, low-density lipoprotein cholesterol, low-density lipoprotein cholesterol, systolic blood pressure, diastolic blood pressure, fasting plasma glucose, insulin resistance index, and insulin and uric acid. **(B)** The quantile-quantile plot for genomic associations of Gamma-glutamyl-leucine (Gamma-Glu-Leu) (*N* = 1,062). **(C)** The Manhattan plot for genomic associations of Gamma-Glu-Leu (*N* = 1,062).

Subjects were recruited from our previous cross-sectional survey on MetS in Hangzhou, Zhejiang of China, which was a questionnaire-based epidemiological investigation conducted in 2010 and consisted of 862 patients with MetS and 880 healthy controls. The detailed information for the study population has been previously described (28, 29). Fasting blood samples for each subject were collected and then frozen at −80°C immediately. All subjects undertook a whole-genome SNP genotyping and were further divided into discovery (*n* = 1,157) and validation (*n* = 240) subsets to undertake an untargeted metabolomics analysis. After quality control of genotyping and metabolomics data, 1,062 subjects and 227 subjects with good-quality data on Gamma-Glu-Leu measurement and SNP genotyping were included as the discovery and replication samples, respectively. The study overview is shown in Figure 1A.

### Epidemiological investigation and clinical measurements

Using a standardized protocol, a questionnaire-based interview was conducted for each subject by trained investigators. Covariates of demographic characteristics, including age, sex, history of CVDs, type 2 diabetes, hypertension, cancer, liver diseases, kidney diseases, and drug use, were investigated.

Values of body weight, height, waist circumference (WC), hip circumference (HC), systolic blood pressure (SBP), and diastolic blood pressure (DBP) were measured by well-trained assistants using a standardized protocol. Body mass index (BMI) was calculated as the body weight in kilograms divided by the square of the height in meters. WC was determined at the midpoint between the lowest coastal ridge and the upper border of the iliac crest. Blood pressure was measured in a sitting position using a mercury sphygmomanometer. The values of SBP and DBP were reported as the means of three repeat measurements at 30 s intervals.

Serum uric acid (UA), total triglyceride (TG), total cholesterol (TC), high-density lipoprotein cholesterol (HDL-C), low-density lipoprotein cholesterol (LDL-C), and fasting insulin were measured using biochemical auto-analyzers. Fasting plasma glucose (FPG) was analyzed by the glucose oxidase method with a Beckman glucose analyzer. The homeostatic model assessment for insulin resistance (HOMA-IR) was calculated as fasting insulin (μU/L) × fasting glucose (nmol/L)/22.5.

### Gamma-glutamyl-leucine measurement

Serum Gamma-Glu-Leu measurement data was retrieved from our previous untargeted metabolomics study, which was conducted using the Agilent 1290 Infinity coupled with 6545 Q/TOF-MS system (Agilent Technologies, Santa Clara, CA, USA) under the positive and negative ion modes. The chromatographic separation was performed with the Waters BEH C8 analytical column (100 mm × 2.1 mm, 1.7 μm). The mobile phase consisted of acetylene solution (B) and water (A) (both contain 0.1% fore lime acid) in the positive ion mode or methanol solution (B) and water (A) (both contain 10 mmol/L ammonium acetate) in the negative ion mode. Detailed experimental conditions, data acquirement, data preprocessing, and data quality control have been previously described (17, 20). In total, 1,793 ion features were detected in the 240 subjects and 2,238 ion features were detected in the 1,157 subjects. Metabolites were identified according to the public metabolomics databases (The Human Metabolome Database and METLIN) and confirmed using available in-house reference compounds (17).

### Genotyping, imputation, and quality control

Single nucleotide polymorphism genotyping was performed using Illumina Human-OmniExpress 760 k chips (Illumina, San Diego, CA, USA) (28). The staff who performed the DNA analysis were not aware of the clinical status of the subjects. The initial genetic quality control was conducted by removing sex discrepancies, ethnic outliers, probable relatives, and those with a call rate < 0.95 and excessive genome-wide heterozygosity. SNPs with minor allele frequency < 0.05, Hardy-Weinberg equilibrium *P* < 1 × 10^–4^, call rate < 0.95, and not in autosomal chromosomes were excluded. The University of Michigan imputation server was used to complete the imputation (30). Before imputation, all alleles were aligned to the forward strand of build 37 and converted to VCF files. The genotype phasing was conducted using EAGLE with the 1000 Genomes Project Phase 3 Version 5 of EAS as the reference panel. Following imputation, SNPs with poor imputation quality (*r*^2^ < 0.3) were filtered. Consequently, 40,001,312 SNPs remained.

### Statistical analysis

The normality of variables was visually checked using a Q-Q plot. Continuous variables were reported as mean (standard deviation) or median (interquartile range) and were compared using the Student’s *t*-test or Mann–Whitney *U* test. Categorical variables were reported as numbers (percentages) and were compared using the Chi-square test.

Genome-wide association study analyses were conducted using linear regressions under an additive genetic model implemented in PLINK2. Before analyses, for Gamma-Glu-Leu quantification data, inversed-normalized residuals adjusted for age, sex, and the first two genetic principal components were calculated using the R package “GenABEL.” The significance threshold for GWAS was set at *P* < 5 × 10^–8^. The suggestive threshold for GWAS was set at *P* < 5 × 10^–5^. A clump procedure implemented in PLINK was run for all suggestive GWAS signals (1 Mb, *R*^2^ < 0.5). The clumped SNPs were then selected for the replication stage. SNPs with *P* < 0.05 and consistent effect directions were considered successfully replicated. A meta-analysis was performed using the inverse-variance model using the METAL software. The Q-Q and Manhattan plots were drawn using the R package “qqmen.” The regional plots for associations between SNPs and Gamma-Glu-Leu were drawn using Locuszoom based on the Asian population of the 1000 Genome Project. SNPs were annotated with available website databases, including PhenoScanner, the GWAS catalog, GTEx, SNiPA, and HaploReg. The detailed information is shown in Supplementary Table 1.

One-sample MR analyses were used to evaluate the causal relationships between Gamma-Glu-Leu and the cardio-metabolic risk factors, including the main components of the metabolic syndrome: obesity-related traits (BMI, WC, and W/H), lipid traits (TG, TC, LDL-C, and HDL-C), blood pressure (SBP and DBP), and glycemic traits (FPG, HOMA-IR, and insulin). Given that hyperuricemia has been previously suggested to be involved in the MetS, and may be an independent predictor of MetS (31), the UA was also included as a cardio-metabolic risk factor in this study. SNPs that were independently and consistently associated with Gamma-Glu-Leu were used to construct an additive weighted gene risk score (wGRS) as the IV of Gamma-Glu-Leu. The formula is as follows: β means the effect size of the association between the SNP and Gamma-Glu-Leu; *N* means the number of risk alleles; i means the number of instrumental SNPs.


$$\text{w}\,G\,R\,S=\frac{\sum_{1}^{i}\beta \,\text{i}\times \text{N}}{\text{i}\times \sum_{1}^{i}\beta \,\text{i}}$$

To verify the consumption of MR, the F statistics were calculated to evaluate the strength of the IV. The detailed MR analysis process has been provided in Supplementary Figure 1. First, a linear regression was performed to assess the association between wGRS and Gamma-Glu-Leu (βZX); then, the associations of the wGRS with the metabolic risk factors were examined (βZY); and finally, observational estimates of the relationships between Gamma-Glu-Leu and the metabolic risk factors were calculated (βXY). The causal estimates were assessed using the Wald-type estimator. All regression models were adjusted for age and sex. In addition, to evaluate horizontal pleiotropy effects on the results, we calculated the relationships between genetically predicted Gamma-Glu-Leu levels and potential confounding factors (age and gender). By querying the Phenoscanner V3 and GWAS catalog, we also assessed if the identified Gamma-Glu-Leu-associated SNPs were associated with any secondary phenotypes. The statistical significance was set at *P* < 0.05. A two-tailed test was used for all statistical analyses in this study. All statistical analyses were performed using the R version 4.0.3 software.

## Results

### Subject characteristics

A total of 1,062 subjects and 227 subjects were included in the discovery stage and replication stage, respectively. The average age in the discovery stage and validation stage were 57.4 ± 0.3 and 62.4 ± 0.8 years, respectively. The proportions of male and female were 49.9% and 61.2%, respectively. Compared with subjects in the discovery stage, subjects in the validation stage were more likely to be male and older and had a lower BMI and higher WC, and W/H ratio (*P* < 0.05). There was no significant difference in SBP, DBP, TG, TC, UA, HDL-C, LDL-C, insulin, fasting glucose, and HOMA-IR (Table 1).

**TABLE 1:** Characteristics of subjects in genome-wide association study (GWAS) analyses.

	Discovery stage (*N* = 1,062)	Replication stage (*N* = 227)	*P*
Men, *N* (%)	530 (49.9)	139 (61.2)	< 0.01
Age (year)^†^	57.4 (0.3)	62.4 (0.8)	< 0.01
BMI (kg/m^2^)^†^	24.46 (0.11)	23.86 (0.21)	0.01
WC (cm)^†^	82.64 (0.32)	85.9 (0.71)	< 0.01
W/H ratio^†^	0.87 (0.002)	0.9 (0.004)	< 0.01
SBP (mmHg)^†^	140.6 (0.71)	141.48 (1.68)	0.63
DBP (mmHg)^†^	83.56 (0.40)	82.8 (0.90)	0.44
TG (mmol/L)*	1.66 (1.31)	1.61 (1.15)	0.34
LDL-C (mmol/L)^†^	2.16 (0.02)	2.14 (0.05)	0.71
TC (mmol/L)^†^	4.74 (0.03)	4.69 (0.07)	0.46
Uric acid (umol/L)*	5.75 (0.01)	5.76 (0.02)	0.47
HDL-C (mmol/L)^†^	1.5 (0.01)	1.51 (0.02)	0.80
Insulin (mU/L)*	3.9 (3.4)	3.6 (3.2)	0.12
Glucose (mmol/L)*	5.14 (1.12)	5.16 (1.1)	0.25
HOMA-IR*	0.92 (0.96)	0.86 (0.98)	0.33

*Data was presented as median (interquartile range).

^†^Data was presented as mean (standard deviation).

BMI, body mass index; WC, waist circumference; W/H ratio, waist circumference to hip circumference ratio; SBP, systolic blood pressure; DBP, diastolic blood pressure; TG, triglycerides; HDL-C, high density lipoprotein cholesterol; TC, total cholesterol; LDL-C, low-density lipoprotein cholesterol; HOMA-IR, homeostasis model assessment of insulin resistance.

### Two-stage genome-wide association study analyses for Gamma-glutamyl-leucine

**FIGURE 2:**
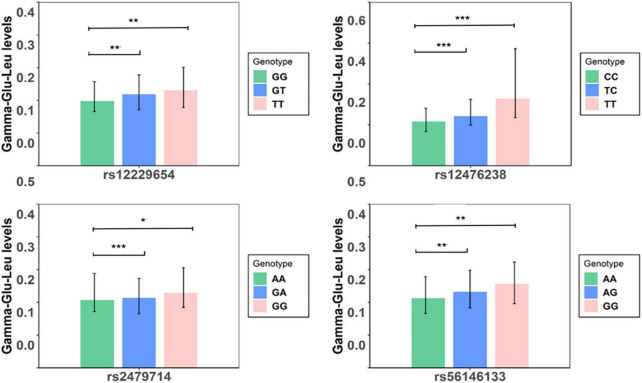
Serum levels of Gamma-glutamyl-leucine (Gamma-Glu-Leu) in subjects with different genotypes (*N* = 1,062). Symbol *denotes that serum levels of Gamma-Glu-Leu were significantly different between groups. ***P-value < 0.001;**P-value < 0.01; *P-value < 0.05.

**FIGURE 3:**
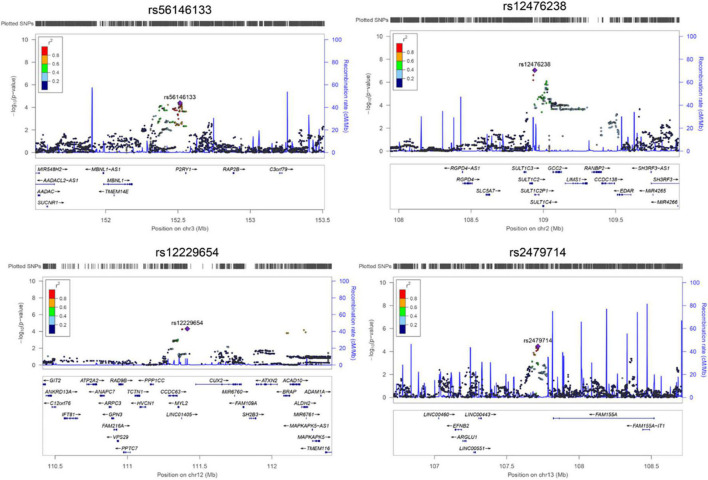
The regional plot for associations between four single nucleotide polymorphisms (SNPs) and Gamma-glutamyl-leucine (Gamma-Glu-Leu) levels (*N* = 1,062). Single nucleotide polymorphism position was based on hg19. Regional plot was drawn using Locuszoom based on the Asian population of the 1000 Genome Project.

In the discovery stage, GWAS analyses showed that the genomic inflation factor for Gamma-Glu-Leu was 1.004, indicating no evidence of population stratification. The Q-Q and Manhattan plots were shown in Figures 1B,C. No significant GWAS signal was found to be associated with Gamma-Glu-Leu (*P* < 5 × 10^–8^, *N* = 1,062). A total of 36 suggestive SNPs were identified to be associated with Gamma-Glu-Leu (*P* < 5 × 10^–5^, Supplementary Table 2). The association between rs12476238 on chromosome 12 and Gamma-Glu-Leu had the lowest *P*-value (*P* = 8.99 × 10^–8^). In the replication stage, four SNPs were successfully replicated to be associated with Gamma-Glu-Leu (Table 2), including rs12476238, rs56146133, rs2479714, and rs12229654. Figure 2 shows that the T allele of rs12229654, the T allele of rs12476238, the G allele of rs2479714, and the G allele of rs56146133 were associated with higher serum Gamma-Glu-Leu levels. The regional plots for the four Gamma-Glu-Leu-associated SNPs are shown in Figure 3, and rs56146133, rs12476238, rs12229654, and rs2479714 can be mapped to the genes of P2RY1, SULTIC2P, MYL2, and FAM155A, respectively.

**TABLE 2:** Four single nucleotide polymorphism (SNPs) that associated with Gamma-Glu-Leu in the two-stage genome-wide association study (GWAS) analyses.

SNP	CHR	POS	REF	ALT	A1	Gene hit	MAF	Discovery stage			Validation stage			*P*-meta
										
								BETA	SE	*P*	BETA	SE	*P*	
rs12476238	2	108940336	T	C	T	SULT1C2P1	0.11	−0.38	0.07	8.99E-08	−0.29	0.14	3.77E-02	1.04E-08
rs56146133	3	152513774	A	G	G	P2RY1	0.22	0.21	0.05	4.01E-05	0.29	0.11	1.09E-02	1.62E-06
rs2479714	13	107715021	G	A	G	FAM155A	0.75	−0.22	0.05	3.61E-05	−0.25	0.11	2.95E-02	3.12E-06
rs12229654	12	111414461	T	G	G	LINC01405	0.25	−0.21	0.05	4.85E-05	−0.28	0.10	7.33E-03	1.49E-06

SNP, single nucleotide polymorphism; CHR, chromosome; POS, position (hg19); REF, reference allele; ALT, alternative allele; MAF, minor allele frequency; A1, minor allele; SE, standard error.

After searching the available SNP annotation databases, rs12229654 was reported to be associated with Gamma-glutamyl transpeptidase (GGT), alcohol consumption behavior (drinker/non-drinker status), quantity of drinks, HDL-C levels, BMI, glycemic traits, and 1 h glucose tolerance levels, specifically in East Asian populations (*P* < 5 × 10^–8^). Out of them, rs12229654 showed the most significant association with Gamma glutamyl-transpeptidase levels (the effect size for the risk allele G: Beta = 0.019, S.E. = 0.0007, *P* = 9.00 × 10^–58^). rs12476238 was reported to be associated with the gene expression of GGC2 and DNA methylation of cg02082929 in the whole blood (*P* < 5 × 10^–8^). In HaploReg, this SNP showed associations with histone modification and DNase from multiple tissues, as well as a motif (BDP1) change. rs56146133 was found to be associated with the gene expression of MBNL1 and the DNA methylation of cg16754766 and cg14921522 in the whole blood (*P* < 5 × 10^–8^). rs2479714 was reported to have associations with two CpG markers cg13810695 and cg05124117 in the whole blood (*P* < 5 × 10^–8^) (Supplementary Tables 3, 4).

Table 3 presents the associations of the four SNPs with cardio-metabolic risk factors in this study. All four SNPs had marginal associations with at least one metabolic trait (5 × 10^–5^ < *P* < 0.05). Notably, rs56146133 was associated with all the listed metabolic traits except for HDL-C, LDL-C, TC, and UA, and rs12229654 was associated with BMI, WC, W/H ratio, SBP, DBP, TG, and UA (5 × 10^–5^ < *P* < 0.05). rs12476238 was associated with BMI and DBP (5 × 10^–5^ < *P* < 0.05). The effect directions for all associations between the four SNPs and metabolic risk factors were consistent with their associations with Gamma-Glu-Leu levels.

**TABLE 3:** Associations of Gamma-glutamyl-leucine (Gamma-Glu-Leu)-associated single nucleotide polymorphism (SNPs) with the cardio-metabolic risk factors (*N* = 1,062).

Traits	rs12229654			rs56146133			rs12476238			rs2479714		
				
	Beta	SE	*P*	Beta	SE	*P*	Beta	SE	*P*	Beta	SE	*P*
BMI	–0.58	0.18	**0.001**	0.37	0.19	**0.049**	0.55	0.25	**0.030**	–0.22	0.19	0.252
WC	–1.75	0.51	**0.001**	1.49	0.53	**0.005**	1.30	0.72	0.073	–1.17	0.53	**0.027**
W/H	–0.01	0.03	**0.003**	0.01	0.003	**0.007**	0.01	0.004	0.14	–0.01	0.003	0.075
SBP	–3.20	1.10	**0.004**	3.34	1.16	**0.004**	2.08	1.55	0.181	–2.23	1.15	0.054
DBP	–1.62	0.64	**0.012**	2.21	0.67	**0.001**	1.96	0.89	**0.028**	–0.73	0.66	0.274
TC	–0.10	0.05	**0.03**	0.02	0.47	0.65	0.02	0.06	0.75	–0.07	0.05	0.127
LDL-C	–0.06	0.04	0.104	–0.01	0.04	0.82	–0.01	0.05	0.83	–0.05	0.04	0.144
TG	–0.03	0.01	**0.018**	0.03	0.01	**0.011**	0.03	0.02	0.097	–0.005	0.01	0.720
UA	–0.03	0.01	**0.018**	0.01	0.01	0.247	0.02	0.02	0.251	–0.02	0.01	0.136
HDL-C	0.01	0.02	0.776	–0.02	0.02	0.300	0.03	0.03	0.320	–0.004	0.02	0.845
Insulin	–0.20	0.17	0.243	0.35	0.17	**0.044**	0.42	0.23	0.069	–0.28	0.17	0.100
FPG	–0.10	0.07	0.162	0.16	0.07	**0.024**	0.12	0.09	0.223	–0.06	0.07	0.408
HOMA-IR	–0.07	0.04	0.049	0.12	0.04	**0.001**	0.12	0.05	**0.014**	–0.06	0.04	0.090

Values of TG were log transformed. Values of UA, FPG, HOMA-IR, and insulin were natural logarithm transformed. The beta was adjusted for age, sex and the first two genetic components. BMI, body mass index; WC, waist circumference; W/H ratio, waist circumference to hip circumference ratio; SBP, systolic blood pressure; DBP, diastolic blood pressure; TG, triglycerides; HDL-C, high density lipoprotein cholesterol; TC, total cholesterol; LDL-C, low-density lipoprotein cholesterol; HOMA-IR, homeostasis model assessment of insulin resistance; SE, standard error. Values in bold denote *P* < 0.05.

### Causal associations of Gamma-glutamyl-leucine with cardio-metabolic risk factors

**FIGURE 4:**
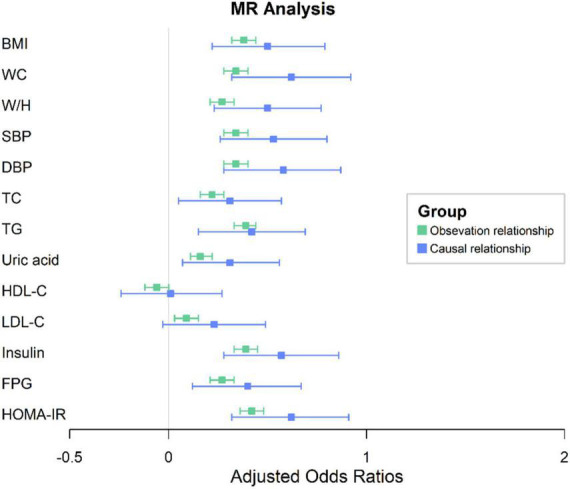
One-sample MR analysis between Gamma-glutamyl-leucine (Gamma-Glu-Leu) and cardio-metabolic risk factors. Colors in green denote the observational relationships. Colors in blue denote the causal relationships. The error bar signifies the beta and 95% CIs. Beta was adjusted for age and sex; BMI, body mass index; WC, waist circumference; W/H ratio, waist circumference to hip circumference ratio; SBP, systolic blood pressure; DBP, diastolic blood pressure; TG, triglycerides; HDL-C, high density lipoprotein cholesterol; TC, total cholesterol; LDL-C, low-density lipoprotein cholesterol; HOMA-IR, homeostasis model assessment of insulin resistance; MR, Mendelian Randomization; CI, confidence interval.

Genetically predicted serum Gamma-Glu-Leu levels were not significantly associated with age and gender (*P* > 0.05, Supplementary Table 5). In Phenoscanner and the GWAS catalog, we also did not find that the identified four SNPs had associations with possible confounding factors (Supplementary Table 3). Therefore, all of them were used to construct a GRS of Gamma-Glu-Leu, which was then used as its IV (Beta = 0.25, S.E. = 0.03, *P* = 1.26 × 10^–14^). The F statistic for this IV was 46.39, suggesting that it was sufficient for the MR analyses. After adjustment for age and sex, observational associations showed that higher levels of Gamma-Glu-Leu were significantly associated with all listed metabolic risk factors except for HDL-C (*P* < 0.05). For the causal estimates, similarly, higher genetically determined levels of Gamma-Glu-Leu were positively associated with all listed metabolic risk factors except for HDL-C and LDL-C (Figure 4 and Supplementary Table 6, *P* < 0.05). Among these outcomes, higher Gamma-Glu-Leu displayed the most significant causal relationship with high HOMA-IR [Beta: 0.62 (95% CI: 0.32–0.91), *P* = 3.6 × 10^–5^].

## Discussion

In this study, we first reported that four SNPs were associated with serum Gamma-Glu-Leu levels in the East Asian population, including rs12476238, rs56146133, rs2479714, and rs12229654. Furthermore, each of these SNPs was nominally associated with at least one cardio-metabolic risk factor. MR analyses revealed that higher serum levels of Gamma-Glu-Leu were causally linked to the risk of cardio-metabolic factors. Overall, our study provides the causal evidence between Gamma-Glu-Leu levels and the metabolic risks. Further mechanistic research is needed to explore how this metabolite affects metabolic abnormality.

rs12229654 is located in 12q24.11 and is within a lncRNA gene (LINC01405). Its upstream gene is called MYL2, encoding a regulatory light chain associated with the cardiac myosin β (or slow) heavy chain. Its downstream gene is called CUX2, encoding a protein that contains three CUT domains and a homeodomain. Both domains are DNA-binding motifs. Previous GWAS studies have reported that rs12229654 was associated with GGT, BMI, HDL-C, glycemic traits, alcohol consumption behavior and quantity of drinks, and the risk of hypertension and T2D in the East Asian population (32–36). Consistently, our study found that rs12229654 was associated with Gamma-Glu-Leu (a metabolic product of GGT), BMI, WC, W/H ratio, TG, TC, and blood pressure. GGT is a liver enzyme involved in GSH metabolism, and the liver is the main alcohol-detoxification organ. The associations among rs12229654, GGT, as well as its related metabolite (Gamma-Glu-Leu), alcohol consumption behavior and amount of drinks suggested that the observed associations of rs12229654 with serum Gamma-Glu-Leu and cardio-metabolic risk factors may be partly explained by drinking behavior and its followed abnormal liver function (12, 37). Of note, allele G of rs12229654 only showed up in the East Asian population (MAF = 0.15 in the 1000 Genome Project) but not in other ethnic groups. Since most annotation databases on SNP regulatory elements were based on the European population, little annotated information on this SNP was reported. Future functional studies are required to uncover the mechanism of rs12229654 in metabolic disorders.

rs12476238 is located in 2q12.3, its mapped gene is a pseudogene called sulfotransferase Family 1C Member 2 (SULT1C2). The Meta-Analyses of Glucose and Insulin-related traits Consortium (MAGIC) previously reported nominal associations between rs12476238 and insulin and HOMA-IR (38). Similarly, a nominal relationship between rs12476238 and HOMA-IR was also reported in this study, indicating that rs12476238 may have an impact on glucose balance. According to the annotation database, rs12476238 is a *trans*-eQTL that can regulate the gene expression of its downstream gene GCC2 (39). GCC2 encodes a membrane protein localized to the *trans*-Golgi network and is related to the vesicular transport between the endosomes and the Golgi. rs12476238 was also associated with CpG marker cg02082929, which is close to the exon of GCC2 (40). In HaploReg, rs12476238 showed regulatory effects on histone modification and DNase in multiple tissues, suggesting that GCC2 may be the target gene of rs12476238. Notably, the frequency of allele T of this SNP was 0.87 in the Asian population of the 1000 genome projects, which was significantly higher than in other populations (EUR: 0.15; AFR: 0.08). Extensive studies are required to verify whether GCC2 links the association between rs12476238 and Gamma-Glu-Leu.

rs56146133 is located in 3q25.2 and can be mapped to the gene for purinergic receptor P2Y1. The product of this gene belongs to the family of G-protein-coupled receptors. Gene Ontology annotations on this gene include G-protein-coupled receptor activity and signaling receptor activity. rs56146133 was associated with the gene expression of MBNL1, which is located ∼330 kb downstream of this SNP. MBNL1 encodes a member of the muscle-blind protein family. This protein is a C3H-type zinc finger protein and modulates alternative splicing of pre-mRNAs. Gloria et al. have reported that MBNL1 activates insulin receptor exon 11 inclusion by enhancing U2AF65 binding and splicing of the upstream intron (41). Alternative splicing regulates developmentally and tissue-specific gene expression programs, disruption of which has been implicated in numerous diseases. In this study, we observed that rs56146133 was associated with Gamma-Glu-Leu and multiple metabolic risk factors, indicating that this gene locus may have a regulatory role on metabolic risk.

rs2479714 is located at 13q33.3 and can be aligned to its nearby gene, FAM155A. This gene has been predicted to be involved in calcium ion import across the plasma membrane. No studies have reported the association of rs2479714 with any traits or diseases, but one gene variant, rs1509091, within 13q33.3 has been identified as having a suggestive relationship with serum pyroglutamine measurement (*P* = 3 × 10^–6^) (42). Pyroglutamine, also known as 5-oxoproline, is an intermediate metabolite in GSH metabolism. The Gamma-glutamyl amino acid that is released into the cytosol can be further catabolized into 5-oxoproline and amino acids by Gamma-glutamyl cyclotransferase (GCT) (43, 44), which suggested that the gene locus of 13q33.3 may be associated with the GSH cycle.

Using identified four SNPs as IV of Gamma-Glu-Leu, we found higher serum Gamma-Glu-Leu levels causally linked to multiple cardio-metabolic risks, which was consistent with previous metabolomics research (17, 45). Gamma-Glu-Leu is partly derived from GSH degradation by GGT, whose elevation may be due to an increased turnover of GSH and an increased GGT activity. It has been demonstrated that an elevated GGT activity and a depletion of GSH are associated with the risk of new onset of MetS and type 2 diabetes (46–49). Additionally, Gamma-Glu-Leu exhibited a negative association with levels of selenoprotein P (50), which is also an important antioxidant substance synthesized in the liver and has a critical role in maintaining the balance of glucose and lipid metabolism (51). Little is known about the pathological function of Gamma-Glu-Leu. Previous experimental studies reported the beneficial effects of Gamma-Glu-Leu and Gamma-glutamyl valine including anti-inflammation and hypoglycemic action; however, most of them were conducted *in vitro* cell models (9, 52, 53). Future interventional studies in human and animal models are required to longitudinally assess the metabolic effects of Gamma-Glu-Leu, and further reveal the molecular mechanisms of its involvement in the pathophysiology of metabolic disturbances.

Our study has some strengths. First, using two-stage GWAS analyses, we first reported the genetic associations of Gamma-Glu-Leu and identified four SNPs to be associated with serum levels of Gamma-Glu-Leu, which provides mechanistic insights into the involvement of Gamma-Glu-Leu in the metabolic disorders. In addition, using the MR approach, we revealed that higher Gamma-Glu-Leu may causally contribute to elevated cardio-metabolic risks.

Several other limitations should also be considered. First, all subjects of this study were derived from East China, thus the findings of this study cannot be generalized to other populations. Second, a few significant SNPs associated with Gamma-Glu-Leu were identified, which may be due to our limited sample size, although this study was the largest GWAS for this metabolite in East China populations. Thirdly, information on lifestyle exposure was not collected in the epidemiological investigation, such as drinking behavior, so its effect on the genetic associations of Gamma-Glu-Leu cannot be analyzed. Fourth, as a well-established cardio-metabolic risk factor, levels of C-reactive protein were not measured in this study, which prevented us from exploring the relationship between Gamma-Glu-Leu and systemic inflammation. Fifth, the instrumental SNPs used to construct the IV did not obtain the GWAS significance threshold, which might cause a weak instrumental bias. However, the F statistics of the IV were larger than 10, indicating that the IV was enough for the MR analyses. Additionally, the gene pleiotropy in the MR analyses cannot be completely avoided.

## Conclusion

Four SNPs (rs12476238, rs56146133, rs2479714, and rs12229654) may regulate the levels of serum Gamma-Glu-Leu. Higher Gamma-Glu-Leu levels are causally linked to the cardio-metabolic risk. Future interventional studies on Gamma-Glu-Leu are required to explain its role in metabolic disorders.

## Data availability statement

The data presented in this study are deposited in the figshare repository, accession number: https://doi.org/10.6084/m9.figshare.20103644.v1.

## Ethics statement

The studies involving human participants were reviewed and approved by the Research Ethics Committee at the School of Medicine, Zhejiang University. The patients/participants provided their written informed consent to participate in this study.

## Author contributions

ML, YZ, and QC: conceptualization. QW, XS, DH, ZC, and JuL: data curation. JiL and JZ: formal analysis. YZ and YX: funding acquisition. QW, DH, ZC, and JuL: investigation. QW, JuL, and XS: methodology. ML and YZ: project administration. YZ: resources and supervision. QW and JZ: software. QW: validation, visualization, and writing – original draft. YZ and XZ: writing – review and editing. All authors approved the final edited version of this manuscript.

## References

[1] Wu SH Liu Z Ho SC. Metabolic syndrome and all-cause mortality: a meta-analysis of prospective cohort studies. *Eur J Epidemiol.* (2010) 25:375–84. 20425137 10.1007/s10654-010-9459-z

[2] Ranasinghe P Mathangasinghe Y Jayawardena R Hills AP Misra A. Prevalence and trends of metabolic syndrome among adults in the Asia-pacific region: a systematic review. *BMC Public Health.* (2017) 17:101. 10.1186/s12889-017-4041-1 28109251 PMC5251315

[3] Alberti KG Zimmet P Shaw J. Metabolic syndrome–a new world-wide definition. A consensus statement from the international diabetes federation. *Diabet Med.* (2006) 23:469–80. 10.1111/j.1464-5491.2006.01858.x 16681555

[4] Candi E Tesauro M Cardillo C Lena AM Schinzari F Rodia G Metabolic profiling of visceral adipose tissue from obese subjects with or without metabolic syndrome. *Biochem J.* (2018) 475:1019–35. 29437994 10.1042/BCJ20170604

[5] Toelstede S Dunkel A Hofmann T. A series of kokumi peptides impart the long-lasting mouthfulness of matured gouda cheese. *J Agric Food Chem.* (2009) 57:1440–8. 10.1021/jf803376d 19170504

[6] Frerot E Chen T. Identification and quantitation of new glutamic acid derivatives in soy sauce by UPLC/MS/MS. *Chem Biodivers.* (2013) 10:1842–50. 10.1002/cbdv.201300150 24130027

[7] Zhang H Kovacs-Nolan J Kodera T Eto Y Mine Y. γ-Glutamyl cysteine and γ-glutamyl valine inhibit TNF-α signaling in intestinal epithelial cells and reduce inflammation in a mouse model of colitis via allosteric activation of the calcium-sensing receptor. *Biochim Biophys Acta.* (2015) 1852:792–804. 10.1016/j.bbadis.2014.12.023 25558818

[8] Guha S Paul C Alvarez S Mine Y Majumder K. Dietary γ-Glutamyl Valine ameliorates TNF-α-Induced vascular inflammation via endothelial calcium-sensing receptors. *J Agric Food Chem.* (2020) 68:9139–49. 10.1021/acs.jafc.0c04526 32786865 PMC8012099

[9] Xing L Zhang H Majumder K Zhang W Mine Y. γ-glutamylvaline prevents low-grade chronic inflammation via activation of a calcium-sensing receptor pathway in 3T3-L1Mouse adipocytes. *J Agric Food Chem.* (2019) 67:8361–9. 10.1021/acs.jafc.9b02334 31339708

[10] Salama SA Arab HH Hassan MH Al Robaian MM Maghrabi IA. Cadmium-induced hepatocellular injury: modulatory effects of γ-glutamyl cysteine on the biomarkers of inflammation, DNA damage, and apoptotic cell death. *J Trace Elem Med Biol.* (2019) 52:74–82. 10.1016/j.jtemb.2018.12.003 30732903

[11] Guha S Majumder K. Comprehensive review of γ-glutamyl peptides (γ-GPs) and their effect on inflammation concerning cardiovascular health. *J Agric Food Chem.* (2022) 70:7851–70. 10.1021/acs.jafc.2c01712 35727887

[12] Kalhan SC Guo L Edmison J Dasarathy S McCullough AJ Hanson RW Plasma metabolomic profile in nonalcoholic fatty liver disease. *Metabolism.* (2011) 60:404–13. 20423748 10.1016/j.metabol.2010.03.006 PMC2950914

[13] Capel F Bongard V Malpuech-Brugère C Karoly E Michelotti GA Rigaudière JP Metabolomics reveals plausible interactive effects between dairy product consumption and metabolic syndrome in humans. *Clin Nutr.* (2020) 39:1497–509. 10.1016/j.clnu.2019.06.013 31279616

[14] Comte B Monnerie S Brandolini-Bunlon M Canlet C Castelli F Chu-Van E Multiplatform metabolomics for an integrative exploration of metabolic syndrome in older men. *EBioMedicine.* (2021) 69:103440. 10.1016/j.ebiom.2021.103440 34161887 PMC8237302

[15] Surowiec I Noordam R Bennett K Beekman M Slagboom PE Lundstedt T Metabolomic and lipidomic assessment of the metabolic syndrome in Dutch middle-aged individuals reveals novel biological signatures separating health and disease. *Metabolomics.* (2019) 15:23. 10.1007/s11306-019-1484-7 30830468 PMC6373335

[16] Saoi M Sasaki K Sagawa H Abe K Kogiso T Tokushige K High throughput screening of serum γ-glutamyl dipeptides for risk assessment of nonalcoholic steatohepatitis with impaired glutathione salvage pathway. *J Proteome Res.* (2020) 19:2689–99. 10.1021/acs.jproteome.9b00405 31483669

[17] Li J Li J Wang H Qi LW Zhu Y Lai M. Tyrosine and glutamine-leucine are metabolic markers of early-stage colorectal cancers. *Gastroenterology.* (2019) 157:257–259.e5. 10.1053/j.gastro.2019.03.020 30885779

[18] Zheng Y Yu B Alexander D Steffen LM Nettleton JA Boerwinkle E. Metabolomic patterns and alcohol consumption in African Americans in the atherosclerosis risk in communities study. *Am J Clin Nutr.* (2014) 99:1470–8. 10.3945/ajcn.113.074070 24760976 PMC4021786

[19] Yang XL Cui ZZ Zhang H Wei XT Feng GJ Liu L Causal link between lipid profile and bone mineral density: a Mendelian randomization study. *Bone.* (2019) 127:37–43. 10.1016/j.bone.2019.05.037 31158506

[20] Wu Q Sun X Chen Q Zhang X Zhu Y. Genetically predicted selenium is negatively associated with serum TC, LDL-C and positively associated with HbA1C levels. *J Trace Elem Med Biol.* (2021) 67:126785. 10.1016/j.jtemb.2021.126785 34015661

[21] Lotta LA Scott RA Sharp SJ Burgess S Luan J Tillin T Genetic predisposition to an impaired metabolism of the branched-chain amino acids and risk of type 2 diabetes: a Mendelian randomisation analysis. *PLoS Med.* (2016) 13:e1002179. 10.1371/journal.pmed.1002179 27898682 PMC5127513

[22] Grover S Del Greco MF Stein CM Ziegler A. Mendelian randomization. *Methods Mol Biol.* (2017) 1666:581–628. 28980266 10.1007/978-1-4939-7274-6_29

[23] Hagenbeek FA Pool R van Dongen J Draisma HHM Jan Hottenga J Willemsen G Heritability estimates for 361 blood metabolites across 40 genome-wide association studies. *Nat Commun.* (2020) 11:39. 10.1038/s41467-019-13770-6 PMC6946682 31911595

[24] Adamski J Suhre K. Metabolomics platforms for genome wide association studies–linking the genome to the metabolome. *Curr Opin Biotechnol.* (2013) 24:39–47. 10.1016/j.copbio.2012.10.003 23102864

[25] Kettunen J Demirkan A Wurtz P Draisma HH Haller T Rawal R Genome-wide study for circulating metabolites identifies 62 loci and reveals novel systemic effects of LPA. *Nat Commun.* (2016) 7:11122. 10.1038/ncomms11122 27005778 PMC4814583

[26] Shin SY Fauman EB Petersen AK Krumsiek J Santos R Huang J An atlas of genetic influences on human blood metabolites. *Nat Genet.* (2014) 46:543–50. 24816252 10.1038/ng.2982 PMC4064254

[27] Sun Y Lu YK Gao HY Yan YX. Effect of metabolite levels on type 2 diabetes mellitus and glycemic traits: a Mendelian randomization study. *J Clin Endocrinol Metab.* (2021) 106:3439–47. 10.1210/clinem/dgab581 34363473

[28] Zhu Y Zhang D Zhou D Li Z Li Z Fang L Susceptibility loci for metabolic syndrome and metabolic components identified in Han Chinese: a multi-stage genome-wide association study. *J Cell Mol Med.* (2017) 21:1106–16. 10.1111/jcmm.13042 28371326 PMC5431133

[29] Liu Y Wu M Ling J Cai L Zhang D Gu HF Serum IGFBP7 levels associate with insulin resistance and the risk of metabolic syndrome in a Chinese population. *Sci Rep.* (2015) 5:10227. 10.1038/srep10227 25984973 PMC4650783

[30] Chen MH Raffield LM Mousas A Sakaue S Huffman JE Moscati A Trans-ethnic and Ancestry-specific blood-cell genetics in 746,667 individuals from 5 global populations. *Cell.* (2020) 182:1198–1213.e14. 10.1016/j.cell.2020.06.045 32888493 PMC7480402

[31] Ali N Miah R Hasan M Barman Z Mou AD Hafsa JM Association between serum uric acid and metabolic syndrome: a cross-sectional study in Bangladeshi adults. *Sci Rep.* (2020) 10:7841. 10.1038/s41598-020-64884-7 PMC7217902 32398834

[32] Kim YJ Go MJ Hu C Hong CB Kim YK Lee JY Large-scale genome-wide association studies in East Asians identify new genetic loci influencing metabolic traits. *Nat Genet.* (2011) 43:990–5. 21909109 10.1038/ng.939

[33] Heo SG Hwang JY Uhmn S Go MJ Oh B Lee JY Male-specific genetic effect on hypertension and metabolic disorders. *Hum Genet.* (2014) 133:311–9. 24142389 10.1007/s00439-013-1382-4

[34] Shim U Kim HN Sung YA Kim HL. Pathway analysis of metabolic syndrome using a genome-wide association study of Korea associated resource (KARE) cohorts. *Genomics Inform.* (2014) 12:195–202. 10.5808/GI.2014.12.4.195 25705158 PMC4330254

[35] Jorgenson E Thai KK Hoffmann TJ Sakoda LC Kvale MN Banda Y Genetic contributors to variation in alcohol consumption vary by race/ethnicity in a large multi-ethnic genome-wide association study. *Mol Psychiatry.* (2017) 22:1359–67. 10.1038/mp.2017.101 28485404 PMC5568932

[36] Wen W Zheng W Okada Y Takeuchi F Tabara Y Hwang JY Meta-analysis of genome-wide association studies in East Asian-ancestry populations identifies four new loci for body mass index. *Hum Mol Genet.* (2014) 23:5492–504. 24861553 10.1093/hmg/ddu248 PMC4168820

[37] Cheng J Joyce A Yates K Aouizerat B Sanyal AJ. Metabolomic profiling to identify predictors of response to vitamin E for non-alcoholic steatohepatitis (NASH). *PLoS One.* (2012) 7:e44106. 10.1371/journal.pone.0044106 23028489 PMC3446974

[38] Dupuis J Langenberg C Prokopenko I Saxena R Soranzo N Jackson AU New genetic loci implicated in fasting glucose homeostasis and their impact on type 2 diabetes risk. *Nat Genet.* (2010) 42:105–16. 10.1038/ng.520 20081858 PMC3018764

[39] Zhernakova DV Deelen P Vermaat M van Iterson M van Galen M Arindrarto W Identification of context-dependent expression quantitative trait loci in whole blood. *Nat Genet.* (2017) 49:139–45. 10.1038/ng.3737 27918533

[40] Bonder MJ Luijk R Zhernakova DV Moed M Deelen P Vermaat M Disease variants alter transcription factor levels and methylation of their binding sites. *Nat Genet.* (2017) 49:131–8. 27918535 10.1038/ng.3721

[41] Echeverria GV Cooper TA. Muscleblind-like 1 activates insulin receptor exon 11 inclusion by enhancing U2AF65 binding and splicing of the upstream intron. *Nucleic Acids Res.* (2014) 42:1893–903. 10.1093/nar/gkt1020 24185704 PMC3919616

[42] Yu B Zheng Y Alexander D Manolio TA Alonso A Nettleton JA Genome-wide association study of a heart failure related metabolomic profile among African Americans in the atherosclerosis risk in communities (ARIC) study. *Genet Epidemiol.* (2013) 37:840–5. 10.1002/gepi.21752 23934736 PMC4079107

[43] Bachhawat AK Yadav S. The glutathione cycle: glutathione metabolism beyond the γ-glutamyl cycle. *IUBMB Life.* (2018) 70:585–92. 10.1002/iub.1756 29667297

[44] Comuzzie AG Cole SA Laston SL Voruganti VS Haack K Gibbs RA Novel genetic loci identified for the pathophysiology of childhood obesity in the hispanic population. *PLoS One.* (2012) 7:e51954. 10.1371/journal.pone.0051954 23251661 PMC3522587

[45] Fall T Salihovic S Brandmaier S Nowak C Ganna A Gustafsson S Non-targeted metabolomics combined with genetic analyses identifies bile acid synthesis and phospholipid metabolism as being associated with incident type 2 diabetes. *Diabetologia.* (2016) 59:2114–24. 10.1007/s00125-016-4041-1 27406814 PMC5451119

[46] Neuman MG Malnick S Chertin L. Gamma glutamyl transferase – an underestimated marker for cardiovascular disease and the metabolic syndrome. *J Pharm Pharm Sci.* (2020) 23:65–74. 10.18433/jpps30923 32310756

[47] Ndrepepa G Colleran R Kastrati A. Gamma-glutamyl transferase and the risk of atherosclerosis and coronary heart disease. *Clin Chim Acta.* (2018) 476: 130–8. 29175647 10.1016/j.cca.2017.11.026

[48] Lee DH Jacobs DR Jr. Gross M Kiefe CI Roseman J Lewis CE Gamma-glutamyltransferase is a predictor of incident diabetes and hypertension: the coronary artery risk development in young adults (CARDIA) study. *Clin Chem.* (2003) 49:1358–66. 12881453 10.1373/49.8.1358

[49] Ruiz-Ramírez A Ortiz-Balderas E Cardozo-Saldaña G Diaz-Diaz E El-Hafidi M. Glycine restores glutathione and protects against oxidative stress in vascular tissue from sucrose-fed rats. *Clin Sci (Lond).* (2014) 126:19–29. 10.1042/CS20130164 23742196

[50] di Giuseppe R Koch M Nothlings U Kastenmuller G Artati A Adamski J Metabolomics signature associated with circulating serum selenoprotein P levels. *Endocrine.* (2019) 64:486–95. 10.1007/s12020-018-1816-9 30448992

[51] Tinkov AA Ajsuvakova OP Filippini T Zhou JC Lei XG Gatiatulina ER Selenium and selenoproteins in adipose tissue physiology and obesity. *Biomolecules.* (2020) 10:658. 10.3390/biom10040658 PMC7225961 32344656

[52] Chen Y Zhang H Mats L Liu R Deng Z Mine Y Anti-inflammatory effect and cellular uptake mechanism of peptides from common bean (*Phaseolus vulga* L.) milk and yogurts in caco-2 mono- and Caco-2/EA.hy926 Co-culture models. *J Agric Food Chem.* (2019) 67:8370–81. 10.1021/acs.jafc.9b03079 31271280

[53] Nongonierma AB Mooney C Shields DC Fitzgerald RJ. Inhibition of dipeptidyl peptidase IV and xanthine oxidase by amino acids and dipeptides. *Food Chem.* (2013) 141:644–53. 10.1016/j.foodchem.2013.02.115 23768405

