# Supplementary material for: Gamma-glutamyl-leucine levels are causally associated with elevated cardio-metabolic risks
Source: Front Nutr. 2022 Nov 24;9:936220. doi: 10.3389/fnut.2022.936220 (PMC9729530; doi:10.3389/fnut.2022.936220)
Supplement: Supplementary file 1 [file Data_Sheet_1.docx]

Supplementary Material

# Supplementary Figures and Tables

## Supplementary Figures

**
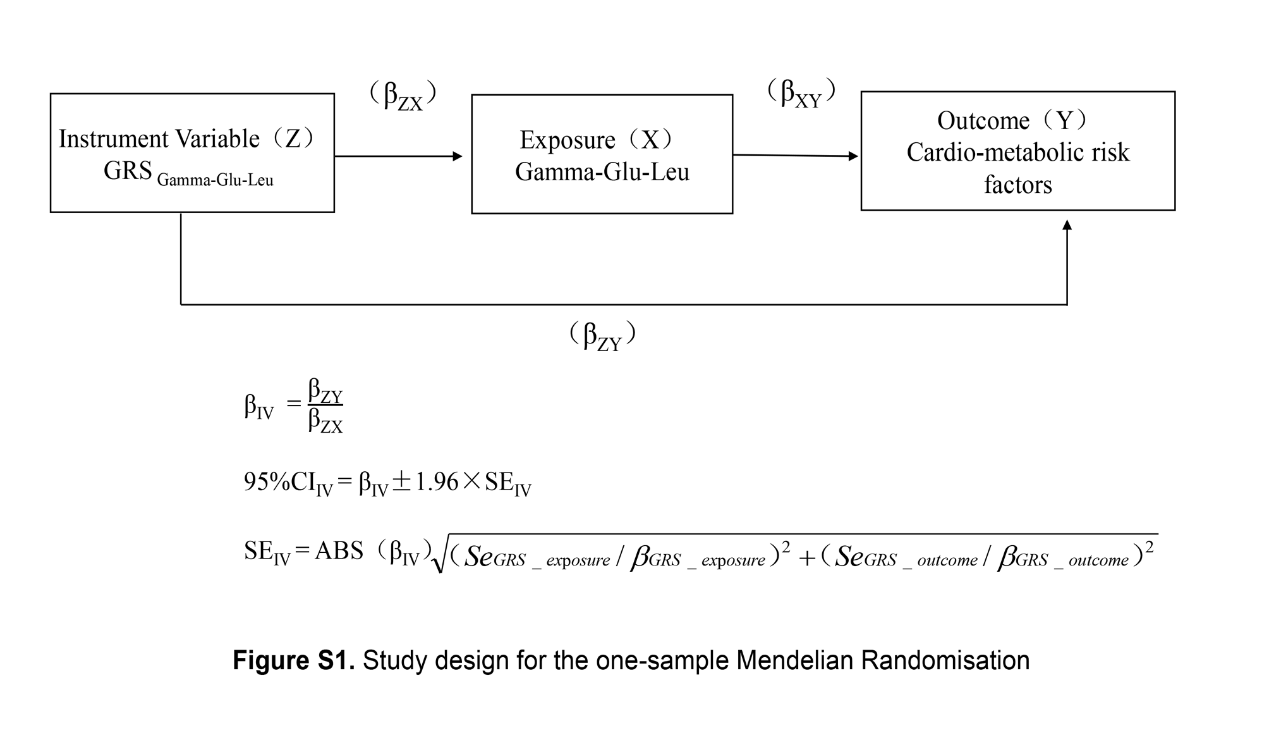
**

**Supplementary Figure 1**. Study design for the one-sample Mendelian Randomization. GRS: gene risk score.

## Supplementary Tables

Table S1. SNP annotation database and tools used in this study.

| Database | Website |
| --- | --- |
| PhenoScanner | http://www.phenoscanner.medschl.cam.ac.uk/ |
| SNiPA | https://snipa.helmholtz-muenchen.de/snipa3/ |
| GWAS catalog | https://www.ebi.ac.uk/gwas/ |
| GTEx | https://www.gtexportal.org/home/ |
| GWAVA | https://www.sanger.ac.uk/sanger/StatGen_Gwava |
| HaploReg | https://pubs.broadinstitute.org/mammals/haploreg/haploreg.php |
| Genecards | https://www.genecards.org/ |

Table S2. 36 SNPs that suggestively associated with serum Gamma-Glu-Leu in the discovery stage (N=1062).

| SNP | CHR | POS | REF | ALT | BETA | SE | P |
| --- | --- | --- | --- | --- | --- | --- | --- |
| rs12476238 | 2 | 108940336 | T | C | -0.38 | 0.07 | 8.99E-08 |
| rs4917504 | 10 | 109347602 | A | G | 0.33 | 0.07 | 2.77E-06 |
| rs356041 | 17 | 6453406 | C | G | 0.40 | 0.09 | 4.96E-06 |
| rs11159586 | 14 | 83743593 | A | G | -0.20 | 0.04 | 6.13E-06 |
| rs58825159 | 4 | 185672945 | A | G | 0.23 | 0.05 | 7.90E-06 |
| rs9523743 | 13 | 93296138 | G | A | 0.26 | 0.06 | 9.27E-06 |
| rs4802456 | 19 | 48768755 | G | A | -0.23 | 0.05 | 9.82E-06 |
| rs7945241 | 11 | 11410851 | G | A | -0.33 | 0.07 | 1.02E-05 |
| rs11126227 | 2 | 69438697 | G | A | -0.21 | 0.05 | 1.23E-05 |
| rs6749474 | 2 | 3313428 | C | A | 0.28 | 0.07 | 1.39E-05 |
| rs8132104 | 21 | 43267796 | G | A | 0.20 | 0.05 | 1.75E-05 |
| rs446684 | 16 | 79222287 | G | A | 0.30 | 0.07 | 1.78E-05 |
| rs16834858 | 1 | 32547587 | T | G | -0.22 | 0.05 | 1.92E-05 |
| rs17138855 | 10 | 16649790 | G | A | 0.19 | 0.04 | 2.16E-05 |
| rs56222178 | 2 | 28932089 | G | A | 0.37 | 0.09 | 2.16E-05 |
| rs1705704 | 8 | 23858386 | A | G | -0.19 | 0.04 | 2.41E-05 |
| rs720893 | 19 | 22426404 | G | A | 0.20 | 0.05 | 2.56E-05 |
| rs3735090 | 7 | 38419878 | T | C | -0.29 | 0.07 | 2.65E-05 |
| rs78616942 | 1 | 54685819 | C | T | -0.26 | 0.06 | 2.69E-05 |
| rs74538992 | 5 | 125362703 | A | G | 0.27 | 0.06 | 2.71E-05 |
| rs1109322 | 2 | 228460817 | A | G | 0.22 | 0.05 | 3.00E-05 |
| rs1660291 | 18 | 62534665 | G | A | 0.23 | 0.05 | 3.01E-05 |
| rs36179089 | 2 | 110253908 | A | G | -0.20 | 0.05 | 3.32E-05 |
| rs752274 | 9 | 101699611 | C | T | 0.25 | 0.06 | 3.66E-05 |
| rs56146133 | 3 | 152513774 | A | G | 0.21 | 0.05 | 4.01E-05 |
| rs1172427 | 13 | 48214274 | A | T | 0.25 | 0.06 | 4.32E-05 |
| rs12220033 | 10 | 6301984 | T | C | -0.27 | 0.07 | 4.40E-05 |
| rs11016987 | 10 | 131668978 | G | A | 0.19 | 0.05 | 4.50E-05 |
| rs17151805 | 7 | 25566545 | T | C | -0.37 | 0.09 | 4.54E-05 |
| rs7950779 | 11 | 6408570 | A | G | -0.23 | 0.06 | 4.66E-05 |
| rs1040018 | 13 | 65419623 | C | T | 0.29 | 0.07 | 4.74E-05 |
| rs80329996 | 12 | 39766618 | C | A | 0.18 | 0.05 | 4.79E-05 |
| rs359941 | 1 | 89976988 | A | G | -0.19 | 0.05 | 4.80E-05 |
| rs2825760 | 21 | 21098110 | T | C | -0.20 | 0.05 | 4.83E-05 |
| rs12229654 | 12 | 111414461 | T | G | -0.21 | 0.05 | 4.85E-05 |
| rs9587284 | 13 | 107711429 | A | G | -0.21 | 0.05 | 5.00E-05 |

SNP, single nucleotide polymorphism; CHR, chromosome; POS, position (hg19); REF, reference allele; ALT, alternative allele; SE, standard error.

Table S3. Annotation information of the four gamma-Glu-Leu-associated SNPs from PhenoScanner.

| SNP | hg19_coordinates | A1 | A2 | Trait | Ancestry | Beta | SE | P | N |
| --- | --- | --- | --- | --- | --- | --- | --- | --- | --- |
| rs12229654 | chr12:111414461 | G | T | Gamma glutamyl transpeptidase | East Asian | 0.0119 | 0.000743 | 9.00E-58 | 24290 |
| rs12229654 | chr12:111414461 | G | T | Alcohol consumption drinkers vs non drinkers | Mixed | — | — | 2.00E-48 | — |
| rs12229654 | chr12:111414461 | G | T | Alcohol consumption male | East Asian | — | — | 8.30E-41 | 1721 |
| rs12229654 | chr12:111414461 | G | T | Alcohol consumption | East Asian | — | — | 3.79E-35 | 5010 |
| rs12229654 | chr12:111414461 | G | T | Alcohol consumption | East Asian | 0.79 | 0.06389 | 4.00E-35 | — |
| rs12229654 | chr12:111414461 | G | T | HDL cholesterol | East Asian | -0.028 | 0.002819 | 3.00E-23 | 26178 |
| rs12229654 | chr12:111414461 | G | T | Esophageal squamous cell carcinoma Esophageal cancer | East Asian | — | — | 3.30E-18 | 1288 |
| rs12229654 | chr12:111414461 | G | T | 1 hour glucose tolerance test | East Asian | — | — | 8.83E-13 | 7696 |
| rs12229654 | chr12:111414461 | G | T | Glycemic traits | East Asian | 0.277 | 0.03877 | 9.00E-13 | — |
| rs12229654 | chr12:111414461 | G | T | Esophageal squamous cell carcinoma Esophageal cancer | East Asian | — | — | 4.07E-09 | 4075 |
| rs12229654 | chr12:111414461 | G | T | Body mass index | East Asian | -0.0341 | 0.005832 | 5.00E-09 | — |
| rs12229654 | chr12:111414461 | G | T | Nonsyndromic cleft lip with cleft palate | East Asian | -0.2357 | 0.04113 | 1.00E-08 | — |
| rs12476238 | chr2:108940336 | C | T | Gene expression GCC2;AC012487.2（Whole blood） | European | — | — | 3.62E-18 | 2116 |
| rs12476238 | chr2:108940336 | C | T | Gene expression GCC2（Whole blood） | European | — | — | 1.47E-14 | 2116 |
| rs12476238 | chr2:108940336 | C | T | Gene expression GCC2;RGPD6;RGPD8;RGPD3;RGPD4;RGPD5 (Whole blood) | Mixed | 0.0379 | 0.00335 | 2.33E-29 | 5257 |
| rs12476238 | chr2:108940336 | C | T | Gene expression GCC2（Whole blood） | European | — | — | 3.03E-74 | 31470 |
| rs12476238 | chr2:108940336 | C | T | DNA methylation cg02082929 (Whole blood) | European | — | — | 1.72E-29 | 3841 |
| rs56146133 | chr3:152513774 | A | G | Gene expression MBNL1(Whole blood) | European | — | — | 4.39E-41 | 31350 |
| rs56146133 | chr3:152513774 | A | G | DNA methylation cg14921522（Whole blood） | European | — | — | 2.72E-19 | 3841 |
| rs56146133 | chr3:152513774 | A | G | DNA methylation cg16754766（Whole blood） | European | 0.3437 | 0.0604 | 1.76E-08 | 837 |
| rs2479714 | chr13:107715021 | A | G | DNA methylation cg13810695 (Whole blood) | European | — | — | 6.16E-38 | 3841 |
| rs2479714 | chr13:107715021 | A | G | DNA methylation cg05124117 (Whole blood) | European | — | — | 4.03E-14 | 3841 |

SNP, single nucleotide polymorphism; A1, effect allele; A2, reference allele; SE, standard error; N, sample size; “—” means the values were not reported

Table S4. Annotation information of the four gamma-Glu-Leu-associated SNPs from HaploReg and GWAVA.

| SNP | CHR | BP | A2 | Allele frequency | | | | HaploReg | | | | | | GWAVA | | |
| --- | --- | --- | --- | --- | --- | --- | --- | --- | --- | --- | --- | --- | --- | --- | --- | --- |
|  |  |  |  | AFR | AMR | ASN | EUR | Enhancer  histone marks | DNAse  Motifs | changed  GRASP QTL | hits  Selected | eQTL hits  NHGRI/EBI | GWAS hits | Region score | TSS score | Unmatched score |
| rs12476238 | 2 | 108940336 | T | 0.08 | 0.39 | 0.87 | 0.15 | 8 tissues | 7 tissues | BDP1 | 1 hit | 2 hits |  | 0.24 | 0.22 | 0.240 |
| rs56146133 | 3 | 152513774 | A | 0.03 | 0.13 | 0.22 | 0.1 |  |  | 5 altered motifs |  |  |  | 0.31 | 0.03 | 0.08 |
| rs2479714 | 13 | 107715021 | G | 0.63 | 0.6 | 0.66 | 0.6 |  |  | 6 altered motifs |  |  |  | 0.26 | 0.08 | 0.02 |
| rs12229654 | 12 | 111414461 | T | 0 | 0 | 0.2 | 0 |  |  | 7 altered motifs |  |  | 6 hits | 0.37 | 0.21 | 0.14 |

SNP, single nucleotide polymorphism; CHR, chromosome; BP: position (hg19); A2, reference allele; AFR, African; AMR, American; ASN, Asian; EUR, European; eQTL: expression quantitative trait loci; GWAS, genome-wide association study; HaploReg, a tool for exploring annotations of the noncoding genome at variants on haplotype blocks. GWAVA, Genome Wide Annotation of variants. The three prediction scores were ranged 0-1, higher scores indicated that variants predicted are more likely to be functional.

Table S5. The associations of genetically predicted Gamma-Glu-Leu levels with gender and age.

| Variables | Beta | S.E. | P |
| --- | --- | --- | --- |
| Age (per one year) | 0.0003 | 0.001 | 0.82 |
| Women Vs Men | 0.0052 | 0.028 | 0.85 |

Table S6. Causal associations between Gamma-Glu-Leu and the cardio-metabolic risk factors (N=1062).

| Outcomes | Observational association | | Causal association | |
| --- | --- | --- | --- | --- |
|  | Beta (95%CI) | P | Beta (95%CI) | P |
| BMI | 0.38(0.32,0.44) | 6.47E-34 | 0.5(0.22,0.79) | 4.75E-04 |
| WC | 0.34(0.28,0.4) | 6.03E-28 | 0.62(0.32,0.92) | 4.80E-05 |
| W/H | 0.27(0.21,0.33) | 1.37E-19 | 0.5(0.23,0.77) | 3.68E-04 |
| SBP | 0.34(0.28,0.4) | 8.42E-31 | 0.53(0.26,0.8) | 1.45E-04 |
| DBP | 0.34(0.28,0.4) | 8.86E-28 | 0.58(0.28,0.87) | 1.04E-04 |
| TC | 0.22(0.16,0.28) | 2.56E-12 | 0.31(0.05,0.57) | 2.28E-02 |
| TG | 0.39(0.33,0.44) | 1.56E-36 | 0.42(0.15,0.69) | 2.68E-03 |
| Uric acid | 0.16(0.11,0.22) | 1.59E-08 | 0.31(0.07,0.56) | 1.28E-02 |
| HDL-C | -0.06(-0.12,0) | 6.03E-02 | 0.01(-0.24,0.27) | 9.34E-01 |
| LDL-C | 0.09(0.03,0.15) | 4.70E-03 | 0.23(-0.03,0.49) | 8.38E-02 |
| Insulin | 0.39(0.33,0.45) | 1.48E-36 | 0.57(0.28,0.86) | 1.43E-04 |
| FPG | 0.27(0.21,0.33) | 6.67E-18 | 0.4(0.12,0.67) | 2.16E-03 |
| HOMA-IR | 0.42(0.36,0.48) | 1.84E-42 | 0.62(0.32,0.91) | 3.60E-05 |

Data was shown as Beta (95%CI). The Beta was adjusted for age and sex. BMI, body mass index; WC, waist circumference; W/H ratio, ratio of waist circumference to hip circumference; SBP, systolic blood pressure; DBP, diastolic blood pressure; TG, triglycerides; HDL-C, high density lipoprotein cholesterol; TC, total cholesterol; LDL-C, low-density lipoprotein cholesterol; HOMA-IR, homeostasis model assessment of insulin resistance; CI, confidence interval.
